# Upregulation of Serum Vascular Endothelial Growth Factor in Patients with Salivary Gland Tumor

**DOI:** 10.1155/2013/740582

**Published:** 2013-07-24

**Authors:** Azadeh Andisheh Tadbir, Bijan Khademi, Mahyar Malekzadeh, Maryam Mardani, Bahar Khademi

**Affiliations:** ^1^Department of Oral and Maxillofacial Pathology, School of Dentistry, Shiraz University of Medical Sciences, Ghom Abad, Ghasrodasht Avenue, Shiraz 71956-15878, Iran; ^2^Department of Otolaryngology, Khalili Hospital, Shiraz Institute for Cancer Research, Shiraz University of Medical Sciences, Shiraz 71348-45794, Iran; ^3^Shiraz Institute for Cancer Research, Shiraz University of Medical Sciences, Shiraz 71348-45794, Iran; ^4^Department of Oral Medicine, School of Dentistry, Shiraz University of Medical Sciences, Ghom Abad, Ghasrodasht Avenue, Shiraz 71956-15878, Iran; ^5^School of Dentistry, Shiraz University of Medical Sciences (International Branch), Azadi Square, Jahad Sazandegi Street, Shiraz 71447-16699, Iran

## Abstract

Neoangiogenesis is essential for tumor development, invasion, and dissemination. The most potent of the cytokines associated with angiogenesis is vascular endothelial growth factor (VEGF). The aim of the present study was to determine VEGF serum level in patients with salivary gland tumor. Using an ELISA kit, the circulating levels of VEGF in sera from 58 patients with salivary gland tumor and 30 healthy controls were assessed. Mean VEGF levels in sera of patients with salivary gland tumors (574.9 ± 414.3) were significantly higher than those in controls (263.9 ± 310.0) (*P* = 0.009). Within the salivary gland tumor group, mean serum VEGF concentration in malignant tumors (*n* = 27) was 727.3 ± 441.8 pg/mL, and that in benign tumors (*n* = 31) was 442.2 ± 343.3 pg/mL. Mean serum VEGF concentration was significantly higher in malignant tumors than in benign tumors (*P* = 0.008) and was higher in benign tumors than in controls (*P* = 0.03). The data in the present study clearly show that VEGF level was consistently upregulated in benign and malignant tumors in comparison to healthy controls. However, the role of VEGF as a prognostic factor in salivary gland tumor and its application in antiangiogenic therapy require further clinical research.

## 1. Introduction

Neoangiogenesis, the development of new vessels from pre-existing ones, is necessary for tumor development, invasion, and dissemination. Angiogenic process not only delivers nutrients for tumor growth but also increases the chance for tumor cells to enter the circulation and metastasis [1].

A variety of signals such as diminished nutrition or oxygen in tissue or genetic changes in oncogenes or tumor suppressor genes can start angiogenic process. Different factors produced from stromal and tumor cells such as growth factors, cytokines, enzymes, and adhesion molecules are involved in this process, and the most potent of them is vascular endothelial growth factor (VEGF) [2].

Among the VEGF family, which consists of VEGF-A, VEGF-B, VEGF-C, VEGF-D, and VEGF-F, VEGF-A is a key angiogenic factor and is mostly used to promote angiogenic phenotype by a tumor [3].

VEGF-A is a dimeric glycoprotein with a molecular weight of 34–45 KDa, consisting of two subunits. Increased VEGF expression was seen in different physiologic and pathologic conditions which was associated with hypoxia [4].

VEGF is expressed in several malignant tumors which indicates its importance in angiogenesis process. Overexpression of VEGF was found in body fluids and solid tumors such as brain, colon, breast, kidney, bladder, lung, and oral cancer [5].

However, no investigations have been conducted regarding serum VEGF levels in patients with salivary gland tumor, so the aim of the present study was to determine VEGF serum level in patients with salivary gland tumor.

## 2. Materials & Methods

### 2.1. Subjects

For the purpose of this case-control study, 58 serum samples from the patients diagnosed with salivary gland tumor (24 males, 34 females, age: 44.8 ± 16.6 years) consisting of 31 pleomorphic adenoma, 17 adenoid cystic carcinoma, and 10 mucoepidermoid carcinoma and 30 serum samples from healthy control subjects (12 males, 18 females, age: 44.6 ± 16.6 years) were collected.

Study subjects were the patients admitted to the Department of Otolaryngology, Khalili Hospital, Shiraz University of Medical Sciences, and diagnosed with salivary gland tumor histopathologically.

Control cases were healthy blood donors, who were matched for age and gender. Exclusion criteria for both groups were based on the presence of any systemic disease, use of corticosteroid or nonsteroid anti-inflammatory medication, or a history of malignancy of any type.

All the participants were informed about the research study and agreed to participate by signing an informed consent form. 

### 2.2. Serum Analysis

Serum samples were obtained from clotted blood following the centrifugation at 4°C and storing at −80°C until analysis. VEGF concentrations were measured by Sandwich ELISA, in accordance with the manufacturer's instructions (BMS, GmbH, Germany).

### 2.3. Statistical Analysis

Statistical analysis was performed using Mann-Whitney test. A *P* value less than 0.05 was considered significant.

## 3. Results

Mean serum VEGF levels in malignant tumors, benign tumors, and controls are shown in Table 1. Mean VEGF levels in sera of patients with salivary gland tumors (574.9 ± 414.3) were significantly higher than those in controls (263.9 ± 310.0) (*P* = 0.009). Within the salivary gland tumor group, mean serum VEGF concentration in malignant tumors (*n* = 27) was 727.3 ± 441.8 pg/mL, and that in benign tumors (*n* = 31) was 442.2 ± 343.3 pg/mL. Mean serum VEGF concentration was significantly higher in malignant tumors than in benign tumors (*P* = 0.008) and was higher in benign tumors than in controls (*P* = 0.03).

No relation was found between mean VEGF levels and sex and age of the patients and controls.

## 4. Discussion

Angiogenesis is necessary for tumor growth. It is a multifaceted process which is regulated by a variety of inducers and inhibitors, which are produced by different cell types like stromal cells, endothelial cells, and tumor cells [6]. Different angiogenic factors have been described. VEGF plays an important role in both pathologic and physiologic conditions [7]. 

VEGF is one of the most important angiogenic factors involved in tumor angiogenesis. It has been suggested that VEGF is a potent mitogen for endothelial cells and can directly induce new blood vessels formation. Concurrently, increased VEGF will enhance microvascular permeability and will lead to plasma protein extravasation (e.g., fibrinogen) into the extravascular area. Then, extra vascular fibrinogen causes clot formation and other proteins like fibronectin are added to fibrin clot and formed a suitable matrix for growth of endothelial cells [8].

Numerous studies have shown that VEGF is involved in tumor progression. VEGF can increase tumor cell extravasation and metastasis through inducing angiogenesis, enhancing vascular permeability, and stimulating immunosuppression [8–10].

Clearly, identification of different biologic markers and understanding of underlying mechanisms may provide useful markers for determining prognosis and effective treatment.

Recently, different functions such as lymphangiogenesis, enhanced vascular permeability, and antiapoptosis activity have been recognized for VEGF which all have a role in tumor progression [10, 11].

VEGF overexpression has been reported in different tumors. Kulapaditharom et al. (2012) found that the most aggressive head and neck squamous cell carcinoma showed the highest VEGF expression. These findings suggest that VEGF has a role in the progression of head and neck squamous cell carcinoma toward an aggressive and invasive phenotype [12].

 Lequerica-Fernández et al. (2007) showed that VEGF can contribute to the progression of salivary gland carcinomas and seems to be associated with lymph node metastasis and worse survival [13].

Recently, Ke-Xiong et al. (2011) suggested that inducible nitric oxide synthase can motivate the expression of VEGF, and its expression status may help evaluate tumor malignancy and prognosis of the patient [14].

A study conducted by Hao et al. (2010) showed that overexpression of VEGF was seen in adenoid cystic carcinoma and its expression was higher in solid form and in advanced-stage group. Also VEGF expression was related to invasion and metastasis and proposed as a prognostic factor [15].

Swelam et al. (2005) suggest that tumor cells of pleomorphic adenoma secrete functional VEGF in different forms that induce their proliferation and differentiation, and VEGF expression is regulated by hypoxic conditions [16].

Recently, circulating VEGF levels have been reported to be a reliable and noninvasive marker of angiogenic activity and were shown to be independent prognostic factors which are correlated with survival in various malignant neoplasms [17], but fewer studies were carried out to evaluate its circulating level. 

Shang et al. showed serum VEGF levels to be significantly elevated in oral cancers compared to control group [18].

Dirix et al. (1996) showed that high serum VEGF was associated with the large size of the primary tumor in advanced colorectal cancer [19].

Khademi et al. (2013) reported that increased preoperative or postoperative serum levels of VEGF can predict disease recurrence and are associated with poor disease-free and overall survival of patients with HNSCC [20].

In the present study, ELISA kit was used to measure serum VEGF concentrations in patients with salivary gland tumor. By reviewing the articles, this study was the first report to do so.

Results of this study indicate a significant increase in VEGF level from healthy controls to benign and malignant tumors.

So VEGF levels can significantly discriminate patients from controls and malignant from benign tumors. Also our results suggest that VEGF levels play a role in the progression of salivary gland tumors which was similar to those of Doi et al. (1999) and de Faria et al. (2011) [21, 22].

## 5. Conclusion

The data in the present study clearly show that VEGF level was consistently upregulated in benign and malignant tumors in comparison to healthy controls. However, the role of VEGF as a prognostic factor in salivary gland tumor and its application in antiangiogenic therapy require further clinical research. 

## Figures and Tables

**Table 1:** Serum VEGF level in benign, malignant salivary gland tumors and normal controls as measured by Sandwich ELISA.

Groups	Mean serum VEGF (pg/mL)	*P* value
Controls	263.9 ± 310 ± 0.0	Controls versus benign tumors *P* = 0.03
Benign salivary gland tumor	442.2 ± 343.3	Controls versus malignant tumors *P* = 0.001
Malignant salivary gland tumor	727.3 ± 441.8	Benign tumors versus malignant tumors *P* = 0.008
